# Short-Term Outcomes of Phage-Antibiotic Combination Treatment in Adult Patients with Periprosthetic Hip Joint Infection

**DOI:** 10.3390/v15020499

**Published:** 2023-02-10

**Authors:** Eugeny Fedorov, Alexander Samokhin, Yulia Kozlova, Svetlana Kretien, Taalai Sheraliev, Vera Morozova, Nina Tikunova, Alexey Kiselev, Vitaliy Pavlov

**Affiliations:** 1 Orthopedics Department, Novosibirsk Research Institute of Traumatology and Orthopedics, 630091 Novosibirsk, Russia; 2 Biotechnology Department, Novosibirsk State Technical University, 630073 Novosibirsk, Russia; 3 Laboratory of Molecular Microbiology, Institute of Chemical Biology and Fundamental Medicine of the Siberian Branch of the Russian Academy of Sciences, 630090 Novosibirsk, Russia; 4 Biostatistics Department, Bekhterev National Medical Research Center for Psychiatry and Neurology of the Ministry of Health of the Russian Federation, 192019 Saint-Petersburg, Russia

**Keywords:** phage therapy, antibiotics, hip joint infection, orthopedics, periprosthetic, PJI, surgery

## Abstract

Implant-associated infections are the most costly problem in modern orthopedics due to the continued increase in the occurrence of antibiotic-resistant bacterial strains that requires the development of new effective antimicrobials. A non-randomized, prospective, open-label, with historical control study on the use of combined phage/antibiotic therapy of periprosthetic joint infection (PJI) was carried out. Forty-five adult patients with deep PJI of the hip joint were involved in the study, with a 12-month follow-up after one-stage revision surgery. Patients from a prospective study group (SG, *n* = 23) were treated with specific phage preparation and etiotropic antibiotics, whereas patients from a retrospective comparator group (CG, *n* = 22) received antibiotics only. The rate of PJI relapses in the SG was eight times less than that in the CG: one case (4.5%) versus eight cases (36.4%), *p* = 0.021. The response rate to treatment was 95.5% (95% confidence interval (CI) = 0.7511–0.9976) in the SG and only 63.6% (95% CI = 0.4083–0.8198) in the CG. The odds ratio for PJI relapse in patients of the SG was 0.083 (95% CI = 0.009–0.742), which was almost 12 times lower than that in the CG. The obtained results support the efficacy of the combined phage-antibiotic treatment of PJI.

## 1. Introduction

Implant-associated infections are the most costly problem in modern orthopedics, with socially significant losses being their most pronounced aspect. Diagnosis and identification of the pathogen, as well as the choice of a rational surgical approach and effective antibacterial therapy, are the key points that determine the outcome of treatment of periprosthetic joint infection (PJI).

Currently, an increase in the occurrence of antibiotic-resistant strains, such as methicillin-resistant *Staphylococcus aureus* (MRSA), methicillin-resistant *Staphylococcus epidermidis* (MRSE), and vancomycin-resistant *Enterococci* (VRE) [1,2,3], exacerbates the PJI problem and is associated with the worsening of treatment outcomes [4,5,6]. The ability of staphylococci and their methicillin-resistant strains to produce lipopolysaccharide biofilms at their adhesion sites, decreases the delivery of antibacterial therapeutic agents to the inflammatory site due to the barrier properties of the bacterial biofilm, thus requiring a significant increase in the minimum inhibitory concentration of antibiotics [7,8,9]. In addition, biofilms can be one of the factors that locally decrease the antibiotic concentration below the effective levels; prolonged exposure to low antibiotic concentrations results in genetic pressure on the bacterial population and accelerated selection of antibiotic-resistant strains, which greatly complicates treatment of infection and often leads to recurrent infections [8,10].

It has been previously shown that if acute PJI caused by antibiotic-resistant strains is treated without implant removal, the rate of successful outcomes is low, approximately 18% [11,12], and two-stage revision, which is the gold standard in most countries, does not completely eradicate the infection [13,14]. The rate of successful outcomes of two-stage treatment for PJI using a daptomycin-loaded spacer and parenteral daptomycin varies from 87.5 to 100% in the case of methicillin-resistant staphylococci [15,16].

One of the promising alternatives is the use of bacteriophages, that are natural viruses capable of infecting and lysing bacteria [17,18,19]. Due to their enzymes, bacteriophages are able to overcome the exopolymeric substrate of biofilms, causing significant changes in the biofilms themselves [20,21,22,23,24] and destroying bacteria inside the biofilm. Experimental studies on the use of bacteriophages as mono- and polyphages in models of PJI, caused by major pathogens, have demonstrated very encouraging results of their application in terms of affecting biofilms and preventing implant colonization [25]. So, bacteriophages are highly sought after as a treatment option in cases of ineffective treatment of PJI, when there is a threat of extremity amputation due to the development of sepsis. A number of clinical cases and pilot studies on the use of phage therapy for the treatment of PJI have been described and reviewed [26,27,28,29,30,31,32]. In these studies, phages were applied during surgery and/or via catheter after surgery. Successful use of phages, both as a last resort monotherapy and as a combination therapy with antibiotics, demonstrated the reasonable possibilities of their application in the treatment of PJI.

Previously, a non-randomized pilot study on the use of combined phage/antibiotic treatment of PJI in patients after hip joint arthroplasty has been carried out in Novosibirsk Research Institute of Traumatology and Orthopedics, Russia [30,33]. The obtained positive results encouraged us to evaluate the efficacy of the studied method of PJI treatment on a relatively large group of patients. Here, a non-randomized, prospective, open-label, with historical control study on the use of combined phage/antibiotic therapy for PJI is described. The original protocol of phage-antibiotic combination therapy was applied in the study group (SG), whereas the approved antibiotic therapy for PJI, according to the current PJI management guidelines, was used in the comparator group (CG). The rate of PJI relapse within 12 months after surgery, and changes in levels of acute inflammatory markers in the early post-operative period after one-stage treatment of PJI, were compared in the two patient groups.

## 2. Materials and Methods

### 2.1. Study Design

The study was designed as non-randomized, prospective, open-label, with historical control in patients with deep periprosthetic hip joint infection. The study was based on comparing the outcomes of two PJI treatment variants: using etiotropic systemic and local antibiotic therapy (CG; historical control), and systemic antibiotic and local phage combination therapy (SG; prospectively recruited), with a commercial staphylococcal bacteriophage preparation pre-selected according to the pathogen susceptibility.

### 2.2. Patients

**Figure 1:**
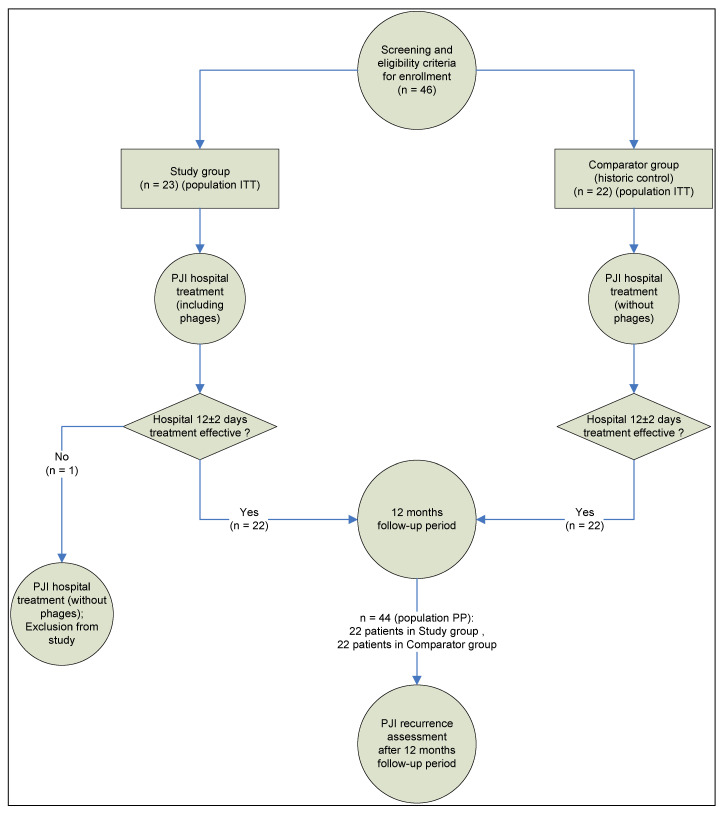
Study design and patient disposition.

The study was based on analysis of outcomes in 45 patients with deep PJI of the hip joint, treated at the Novosibirsk Research Institute of Traumatology and Orthopedics between 2012 and 2018. Informed consent was obtained from all subjects involved in the study. The study design, and patient disposition within intent to treat (ITT) and per protocol populations (PP), are shown in Figure 1. The study was conducted in accordance with the Declaration of Helsinki, and approved by the Ethics Committee of Novosibirsk Research Institute of Traumatology and Orthopedics (protocol 014/17-2 from 25 November 2011).

The **inclusion criteria** for patients in the study were as follows:

(1) Informed consent for surgical treatment.
(2) Patients of any gender aged from 18 to 70 years.
(3) Clinically and laboratory confirmed signs of early post-operative, late chronic, or acute hematogenous PJI, according to previously published classification [34], with the main signs of PJI according to the MSIS criteria [35]: a fistula communicating with the prosthesis; an identified pathogen (in this study, Staphylococcaceae) isolated by culture from two or more separate pre-operative punctures or intraoperative biopsies of periprosthetic tissues; and auxiliary criteria such as acute inflammation markers: an increased erythrocyte sedimentation rate (ESR) and an elevated serum C-reactive protein (CRP) concentration.
(4) Anatomical conditions for cemented endoprosthesis implantation (the possibility of cement pressurization): anatomical bone defects of the proximal femur type I or II according to the Paprosky classification [36] and anatomical bone defects of the acetabulum type I, II, or IIA according to [37].
(5) General somatic condition of the patient being suitable for elective surgery in the form of a one-stage treatment of deep PJI according to the clinical guidelines of 2013 [38] and 2016 [39], including etiotropic antibiotic therapy for up to 12 weeks.
(6) In vitro determined susceptibility of the isolated staphylococcus bacteria to a staphylococcal phage preparation with a titer of at least 10^4^ plaque forming units/mL (PFU/mL).
(7) Eligibility of the patient for the phage therapy protocol within the first ten days after revision surgery.

The **criteria for excluding patients** from the study were as follows:

(1) Patients under the age of 18 and over 70 years.
(2) The absence of the main signs of PJI in the patient according to the MSIS criteria [35].
(3) A lack of anatomical conditions for cemented endoprosthesis implantation (the possibility of cement pressurization): pronounced anatomical bone defects of the proximal femur type IIIA, IIIB, or IV according to Paprosky [36] and anatomical bone defects of the acetabulum type IIB, IIC, III, IIIA, or IIIB [37].
(4) PJI of non-staphylococcal etiology.
(5) Life-threatening conditions requiring urgent surgery before revision arthroplasty.
(6) Lack of sensitivity of isolated *Staphylococcus* spp. to a staphylococcal phage preparation, defined as a bacteriophage titer of less than 10^4^ PFU/mL, before revision arthroplasty.

The **criteria for early withdrawal** of patients from the study:

(1) Life-threatening conditions requiring urgent surgery within 12 ± 2 days after completed revision arthroplasty;
(2) Lack of sensitivity of isolated staphylococcus bacteria to a staphylococcal phage preparation, defined as a bacteriophage titer less than 10^4^ PFU/mL, after completed revision arthroplasty.

### 2.3. Surgical Treatment and Antibiotic Therapy Protocols

Surgical treatment of PJI in patients of both groups was performed in one stage, with implantation of a cemented endoprosthesis.

In the CG, antibiotic (vancomycin) was mixed with bone cement, without vacuum, during implantation of each endoprosthesis component, followed by etiotropic intravenous systemic antibacterial therapy (daptomycin, vancomycin, cefazolin, ciprofloxacin), which was individual for each patient and based on the organism and allergy profile, antibiogram and renal function, according to clinical guidelines for the treatment of periprosthetic infection [39]; a drainage was placed in the postoperative wound for one day.

In the hospital, antibiotic therapy in the CG was prescribed based on the sensitivity of microorganisms to drugs according to the following regimens:

1. Cefazolin at a dose of 2.0 g, three times a day for up to two weeks;
2. Vancomycin at a dose of 1.0 g, twice a day for up to four weeks + daptomycin at a dose of 0.5 g, once a day for up to 3 weeks;
3. Ciprofloxacin at a dose of 0.4 g, twice a day for up to four weeks;
4. Cefazolin at a dose of 2.0 g, three times a day + daptomycin at a dose of 0.5 g, once a day for up to two weeks;
5. Vancomycin at a dose of 1.0 g, twice a day for up to one week + cefazolin at a dose of 2.0 g, three times a day for up to three weeks;
6. Vancomycin at a dose of 1.0 g, twice a day + rifampicin at a dose of 0.6 g, once a day for up to two weeks.

After discharge, patients in this group were prescribed with oral antibiotics administration: ciprofloxacin, at a dose of 0.5 g, twice a day for three to five weeks; or rifampicin, at a dose of 0.3 g, once a day for three months.

### 2.4. Phage Preparations

Three commercial preparations of phage cocktails, produced by Microgen, Russia, were screened: Purified polyvalent pyobacteriophage, Sextaphage^®^ polyvalent pyobacteriophage, and “Staphylococcal bacteriophage”. Before treatment, the PJI pathogen was isolated from two or more separate pre-operative punctures or intra-operative biopsies of periprosthetic tissues and identified by a “VITEK 2 Compact” automatic analyzer (Biomerieux, Craponne, France) or API^®^ strips (Biomerieux, Craponne, France). The susceptibility of the isolated *Staphylococcus* spp. to the above phage preparations was tested using a spot-assay method [40]. In each case, only the “Staphylococcal bacteriophage” was found to be active against the isolated *Staphylococcus* spp. So, next, phage batches were used: H143 and H178 produced by the Perm branch of Microgen (Perm Biomed, Perm, Russia), as well as batches H182 and H184 produced by the Nizhny Novgorod branch of Microgen (IMBIO, Nizhny Novgorod, Russia). 

### 2.5. Original Protocol of Phage-Antibiotic Combination Therapy

In the SG, 6.0 mL of a staphylococcal bacteriophage solution was added during mixing bone cement, without vacuum, before placement of the endoprosthesis components. Such an application of phages has been previously developed by in vitro deposition of phage particles in surface cavities of polymethyl methacrylate implants prepared *ex tempore* [41]. After surgery completion, 20.0 mL of a commercial staphylococcal bacteriophage, whose activity against the pathogen was confirmed in vitro before surgery, was injected into the periprosthetic area daily for ten days through a drainage that was left in the post-operative wound. Simultaneously, etiotropic systemic antibiotic therapy was performed according to the clinical guidelines for the treatment of periprosthetic infection [39]. The placed drainage remained in the post-operative wound for up to ten days for phage therapy. The concentration of bacteriophage administered daily to patients through drains was therapeutic, and amounted to at least 10^5^ PFU/mL. In the case of leakage, the drainage system was removed, and the staphylococcal bacteriophage was administered by puncture.

**Figure 2:**
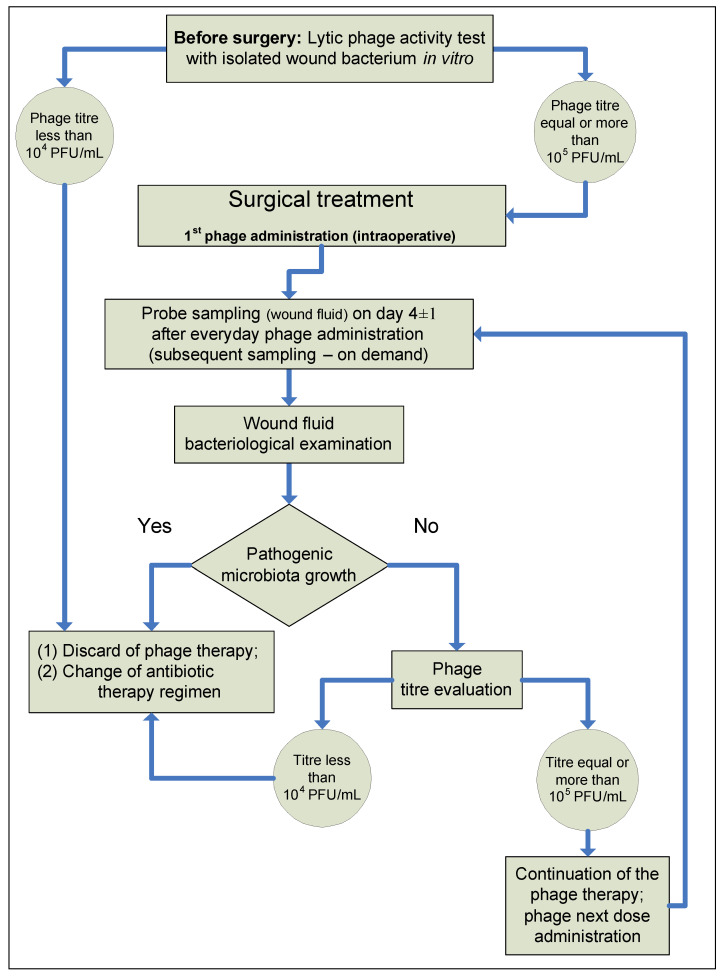
Diagnostic and treatment protocol of combined phage and antibiotic PJI treatment.

In the SG patients, from 5 mL to 10 mL of wound fluid was sampled from a drainage tube on day 4 ± 1 of the postoperative period to monitor the efficacy of phage therapy under hospital conditions. All subsequent wound fluid samples, after day 4 ± 1, were taken on demand. Samples were microbiologically examined and interpreted according to our patented method [42]. A spot-assay method [40] was used to determine the phage titer in the wound fluid. In the case of a negative microbiological test result for a PJI pathogen, the phage titer was determined in vitro in wound fluid using the Appelman method [43]. Detection of a phage titer close to, or higher than, that of a commercial phage in wound fluid indicated a good efficacy of the phage therapy. If the microbiological test of wound fluid from a drainage tube on day 4 ± 1 showed PJI pathogen growth, the phage therapy was considered ineffective. Then, drainage was removed and etiotropic systemic antibiotic therapy alone was used in accordance with the relevant clinical guidelines. The proposed diagnostic and treatment protocol for phage-antibiotic combination therapy is presented in Figure 2.

Because phage therapy was used in the SG in combination with antibiotics (not as monotherapy ), all patients of this group were additionally prescribed etiotropic antibiotic therapy after surgical treatment, for up to twelve weeks, according to the regimens described below. Under hospital conditions (first two weeks), depending on the pathogen, vancomycin was administered as an intravenous drip infusion, at a dose of 1.0 g twice a day, or cefazolin as an intravenous drip infusion at a dose of 2.0 g three times a day, [44]. After discharge, during outpatient treatment, patients were prescribed with oral antibiotics, based on the organism and allergy profile, antibiogram and renal function, according to the following regimens:

1. Ciprofloxacin at a dose of 0.5 g, twice a day for twenty-one days; and rifampicin at a dose of 450 mg, twice a day for ten weeks;
2. Trimethoprim/sulfamethoxazole at a dose of 480 mg, twice a day for one month; then doxycycline at a dose of 100 mg, twice a day for eight weeks.

### 2.6. Evaluation of the Treatment Efficacy

The evaluation was performed simultaneously in two ways, depending on the time since surgery (repeated replacement): evaluation of laboratory and microbiological parameters (ESR, CRP, presence and titers of the PJI pathogen and bacteriophage) in the early post-operative period and the rate of PJI relapse within 12 months after surgery:

- Bacteriological tests: detection and identification of the pathogen was carried out using bacteriological culture. As a study object, we used punctates (in the pre-operative period), biopsies from the inflammation site (open intra-operative biopsy), and wound fluid from drains (in the post-operative period). Microorganisms were identified using a “VITEK 2 Compact” automatic analyzer (Biomerieux, Craponne, France) or API^®^ strips (Biomerieux, Craponne, France).
- Laboratory tests: all patients participating in the study underwent C-reactive protein (CRP) and erythrocyte sedimentation rate (ESR) tests, for laboratory monitoring of an infectious inflammatory process, on days 3 ± 1, 7 ± 1, and 12 ± 2 after surgery. CRP was evaluated with an immunoturbidimetric assay on an Integra Cobas 400 (Roche Diagnostics, Rotkreuz, Switzerland), ESR was evaluated by the Westergren method using an SRS/100II (Greiner Bio-One, Kremsmünster, Austria) automatic analyzer.
- Patient follow-up for PJI relapse: the treatment outcome of PJI was followed up for 12 months after surgery, and the discharge of patients from the hospital using physical examinations and phone interviews recorded as in-person and remote consultations. During surveying patients, their disease history was collected, with focus being placed on pain in the operated hip joint, temperature rises, edema, hyperemia of the post-operative scar or operated hip, possible prescription of antibiotic therapy to the patient, and revision surgery. The treatment efficacy was evaluated based on the rate of PJI relapse according to the criteria of surgical site infection: recovery from PJI considered as no PJI relapse within 12 months; no recovery from PJI considered as PJI relapse within 12 months.

### 2.7. Statistics

All the parameters collected during the study were described using descriptive statistics. The mean (with a 95% confidence interval, CI), standard deviation, minimum and maximum values, median, mode, and quartiles were calculated for interval variables. The category frequencies, percentages, and confidence intervals for frequencies (95% confidence intervals according to the Wilson E.B. method, with continuity correction [45,46]) were calculated for the nominal variables.

Due to the small size of the study population sample, nonparametric descriptive statistics were used to present the results in the article: the median and quartiles (Me [Q1; Q3]). The statistical analysis was performed using the IBM SPSS 25 and R v.4.0.1 software.

***Study endpoints***

***Primary endpoint:*** The rate of periprosthetic infection relapses within 12 months after surgery with a one-stage treatment of PJI.

The analysis results were the rates and percentage of patients with successful and unsuccessful treatment outcomes of PJI in the groups within 12 months. The Fisher’s exact test was used to compare the groups. All *p*-values were calculated as exact *p*-values (not approximated). A 95% CI was calculated using the Wilson E.B. method, with continuity correction [45,46].

***Secondary endpoints:***

(1) The odds ratio for the risk of PJI relapse within 12 months after surgery with one-stage treatment of PJI.

The odds ratio was calculated in addition to the primary study endpoint. For the estimated value, relative and absolute risk estimates were given with a 95% confidence interval, and the effect size was calculated as Phi and Cramer’s V values. All *p*-values were calculated as exact *p*-values (not approximated).

(2) The contents of laboratory markers of the infectious and inflammatory processes (C-reactive protein and erythrocyte sedimentation rate) initially, and on days 3 ± 1, 7 ± 1, and 12 ± 2 after surgery.

Comparisons were performed using the nonparametric Mann–Whitney rank test (for intergroup comparison) and nonparametric Wilcoxon rank test (for intragroup analysis) with Bonferroni correction for multiple comparisons (alpha = 0.1/4 = 0.025). All *p*-values were calculated as exact *p*-values (not approximated for normal distribution).

***Sample size calculation***

In this study, we planned to test the hypothesis of equality of the study method of phage-antibiotic combination therapy in comparison to the comparator method (antibiotic therapy alone):

Null hypothesis, H_0_: *p*_1_ − *p*_0_ = 0
Alternative hypothesis, H_a_: *p*_1_ − *p*_0_ ≠ 0,

where *p*_0_ and *p*_1_ are the response rates to treatment, which is defined as the rate of relapse-free outcomes of PJI treatment within 12 months after one-stage surgery for treatment of PJI in the study method and comparator method groups, respectively. Rejection of the null hypothesis means the lack of equality.

The calculation was performed using the formula for calculating the sample size for the binary endpoint and the hypothesis of equality, assuming that the number of participants in the groups is equal (*n*_0_ = *n*_1_) [page 77, formula 4.7 in [47]]:

$$n_{0}=n_{1}=\frac{{\left(z_{1-\alpha /2}+z_{1-\beta }\right)}^{2}}{{\left(p_{0}-p_{1}\right)}^{2}}\left[p_{0}\left(1-p_{0}\right)+p_{1}\left(1-p_{1}\right)\right]$$

where *z_α/*2*_* and *z_β_* are appropriate values of the z-function for expected type I and II errors, respectively; *p*_0_ and *p*_1_ are the percentage of patients responding to treatment.

In the calculation, the following assumptions were used:

(1) The expected response rate to treatment in the SG (based on our previously reported pilot results) [33]: 91.7% ≈ 91%.
(2) The expected response rate to treatment in the CG (based on the results reported in the literature on hip PJI treatment by antibiotics/DAIR: 58% in the ITT population [48], 60.9% in 8 mg/kg daptomycin [49]): 60%.
(3) Power of the study: 80%.
(4) Significance level: 1% (alpha = 0.1).
(5) Patient number ratio between the groups: 1:1 (*n*_0_ = *n*_1_).

Under the given conditions, the number of patients in each group should be at least:

$$n_{0}=n_{1}=\frac{{\left(1.64+0.84\right)}^{2}\cdot \left(0.91\cdot \left(1-0.91\right)+0.60\cdot \left(1-0.60\right)\right)}{{\left(0.91-0.60\right)}^{2}}=20.601\approx 21$$

Thus, it is necessary to enroll at least 21 patients in each group, or a total of at least 42 patients between the two groups.

## 3. Results

The study was conducted from 2012 to 2018, which was enough time to enroll at least 21 patients in each group. The SG (*n* = 23) included patients who underwent one-stage hip revision surgery due to PJI. The treatment involved cemented implant fixation and the use of the original protocol of etiotropic systemic antibiotic and local phage combination therapies, with phage activity being individually pretested in vitro to the staphylococcus isolated from the patient. The CG (*n* = 22) included patients who underwent one-stage hip revision surgery due to PJI; the treatment involved cemented implant fixation and the use of etiotropic systemic and local antibiotic therapies.

### 3.1. Patients

There were no significant differences between the SG and CG in mean age (56.0 ± 14.56 and 54.4 ± 11.95 years, respectively) and length of hospitalization (27.1 ± 9.64 and 23.6 ± 9.06 days, respectively): *p*-values were 0.376 and 0.188, respectively. Over 50% of patients in both groups displayed late chronic PJI (time interval between one month up to one year after total hip arthroplasty), according to [34] (Table 1).

Identified PJI bacteria were *Staphylococcus* spp. The etiological distribution of PJI pathogens is presented in Table 2. The prevalent causative agents in the SG were methicillin-susceptible *S. aureus* (MSSA) and *S. epidermidis* (MSSE), whereas MSSA was most often detected in the CG.

### 3.2. Primary Endpoint Results

The crosstab for the primary endpoint is as follows (Table 3). The presented data demonstrate that the rate of PJI relapses in the per protocol (PP) population for antibiotic therapy alone (CG) exceeded that of the SG eight-fold, amounting to eight cases (36.4%) in the CG versus one (4.5%) case in the SG (Fisher’s two-sided exact test *p*-value = 0.021). The effect size was moderate: Phi и Cramer’s V values were both equal to 0.394, with both being statistically significant (exact *p*-value = 0.021).

In this case, the response rates to treatment were 95.5% (95% CI = 0.7511–0.9976) in the SG and only 63.6% (95% CI = 0.4083–0.8198) in the CG. Therefore, the difference in response rates to treatment was 31.9% (95% CI = 0.0443–0.5503).

An analysis of the response rates to treatment at the primary endpoint showed that the hypothesis of equality, given that the difference in response rates to treatment between the SG and CG was 31.9% (95% CI for difference = 0.0443–0.5503), was rejected, which indicates a statistically significant difference (Fisher’s two-sided exact test *p*-value = 0.021) between response rates to treatment in patient groups (PP population), given an alpha level of 0.1 used in the study.

### 3.3. Secondary Endpoint Results

The odds ratio, and absolute and relative risk calculations, showed that the risk of PJI relapse (odds ratio) in patients of the SG was less than one (odds ratio = 0.083; 95% CI = 0.009–0.742), and was almost 12 times lower than that in the CG within 12-month follow-up in the PP population (Table 4). The relative risk for the CG was 2.222 (95% CI = 1.393–3.545) and the lower bound of the 95% CI for the CG was higher than the upper bound of the 95% CI in the SG. Thus, an absolute risk for patients in the CG for PJI relapse within 12 months was equal to 2.037, which is two times higher than 1.0.

Regarding laboratory parameters reflecting the infectious inflammatory process, the baseline CRP and ESR values did not differ between the groups (exact *p*-values for CRP and ESR were 0.451 and 0.122, respectively). Descriptive statistics for CRP and ESR within 12 ± 2 days after surgery, and PJI treatment, are presented in Table 5.

Generally, the pattern of CRP and ESR changes in both groups was similar on day 3 ± 1 after surgery, being accompanied by an increase from the baseline values almost 3-fold (*p* = 0.00017) for CRP and 1.5-fold (*p* = 0.004) for ESR in the SG, and 1.67-fold (*p* = 0.009) and 1.15-fold (*p* = 0.616) for CRP and ESR, respectively, in the CG. Further, the median CRP and ESR in the groups decreased, but in different ways: the CRP content decreased rapidly, while the ESR level reduced slowly.

**Figure 3:**
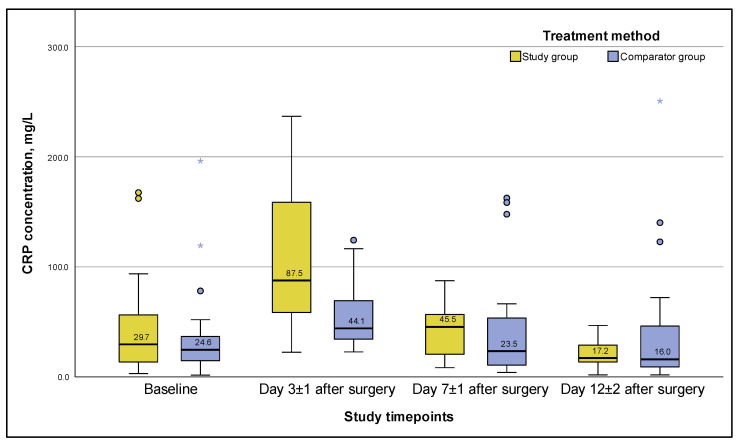
C-reactive protein concentration during PJI treatment for 12 ± 2 days after surgery (ITT population). *Note:* whiskers are based on 1.5 interquartile range, circles and asterisks are outliers.

For example, the CRP level in the SG rapidly decreased up to the end of hospitalization (Figure 3), amounting to 17.2 [13.61; 28.85] mg/L on day 12 ± 2 after surgery, which was a 1.7-fold decrease, significantly less than the baseline value of 29.8 [13.62; 56.40] mg/L (*p*-value = 0.023).

A similar trend in changes in the CRP level was observed in the CG (Figure 3), but with a less pronounced increase in the median and peak values on day 3 ± 1, and a 1.86-fold final decrease to 16.0 [9.06; 46.22] mg/L from the baseline value on day 12 ± 2, with the latter being statistically insignificant (*p*-value = 0.424).

Significant intergroup differences in the CRP content, given the alpha level = 0.025 adjusted for multiple comparisons, were noted only on day 3 ± 1: the medians in the SG and CG differed by almost a factor of two, amounting to 86.8 [57.99; 158.66] and 44.1 [34.40; 69.32] mg/L, respectively (*p* = 0.003). In the following days, up to day 12 ± 2, there were no statistically significant differences in the CRP level between the groups, despite that a two-fold trend in the CRP median difference on day 7 ± 1 was recorded (*p*-value = 0.114).

**Figure 4:**
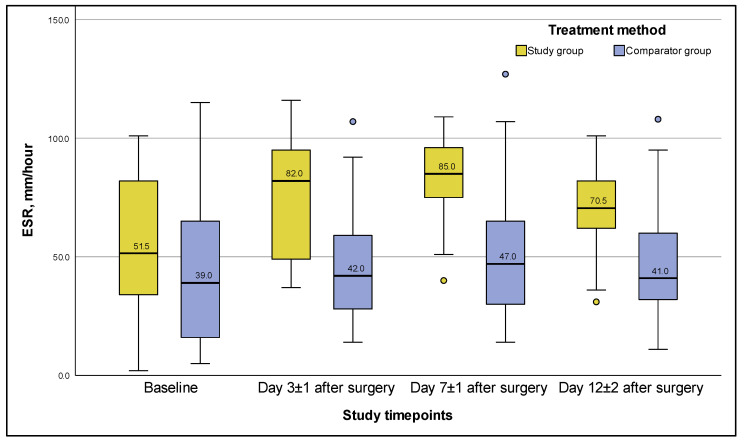
ESR values during PJI treatment for 12 ± 2 days after surgery (ITT population). *Note:* whiskers are based on 1.5 interquartile range, circles are outliers.

The median ESR levels in the CG remained unchanged throughout the entire period of hospitalization (Figure 4), lacking any significant differences between themselves; and even on day 12 ± 2, they had no significant differences from the baseline values (*p*-value = 0.550).

In the SG, the ESR level increased 1.5-fold (*p* = 0.004) from the baseline level by day 3 ± 1 (Figure 4); between day 3 ± 1 and day 7 ± 1, the difference (adjusted for multiple comparisons) was insignificant (*p* = 0.074); by day 7 ± 1, the ESR level was significantly higher than on day 12 ± 2 (*p* = 0.00012); but by the end of hospitalization, on day 12 ± 2, the ESR level did not differ significantly from the baseline value, and amounted to 70.5 [62.00; 82.00] mm/h (*p*-value = 0.061)

### 3.4. Microbiological Test Results

Before using the phage preparation, its efficacy was tested in vitro against the staphylococcal strain isolated from the patient. For all patients from the SG, the preparation was effective with a titer of 10^5^–10^6^ PFU/mL.

To control the efficacy of phage-antibiotic combination therapy under surgical hospital conditions, all patients of the SG (*n* = 23) (ITT population) had a microbiological test of wound fluid on day 4 ± 1 after hip revision surgery. In 22 patients, no pathogenic microorganisms were present on day 4 ± 1, and the bacteriophage titer was 10^2^–10^4^ PFU/mL (Table 6). In the SG (ITT population) on day 4 ± 1, the bacteriophage titer at 10^4^ PFU/mL was only in five patients, the bacteriophage titer was an order of magnitude lower in eight cases, and the titer was 10^2^ PFU/mL in nine cases.

In one patient from the SG (patient number 97182, ITT population), the main PJI pathogen in the wound changed on the fourth day after surgery: MRSE and *Proteus mirabilis* were detected, which was regarded as failure of the phage therapy, which manifested in the absence of a bacteriophage titer and a change in the etiologically significant pathogen. This patient was excluded from the PP study population and then was treated according to the CG protocol.

### 3.5. Adverse Events and Patients Withdrawal

There were no adverse events in the study except for two patients (numbers 89457 and 93001, see Table 6) in the SG with a febrile temperature after administration of the phage preparation. These events were considered to be adverse drug reactions (ADR), definitely related with the phage preparation administration. In these cases, ADRs were moderate and completely resolved during the inpatient period, without additional therapy. All ADRs did not require phage therapy to be withdrawn or patient exclusion from the study.

Only one patient was excluded from the SG (PP population) due to phage therapy failure (more details on this withdrawal case are presented in Section 3.4) and switched to the same treatment as in the CG (see Figure 1). There were no withdrawals in the CG.

## 4. Discussion

In our original protocol of PJI treatment, one of the main requirements for achieving a good clinical and microbiological result is maintenance of an effective therapeutic bacteriophage titer, which is achieved by injection of bacteriophage solutions into the periprosthetic area and measurement of the bacteriophage titer in wound fluid from periprosthetic drains by the Appelman method [43]. For this purpose, we developed a method for monitoring the microbiological efficacy of bacteriophages in the treatment of PJI “A method for assessing the efficacy of phage therapy in the treatment of surgical infections”, patent RU 2624511 C1 of 04 July 2017 [42]. In short, this method suggests that PJI patients are pre-operatively tested for sensitivity of an isolated staphylococcal strain to staphylococcal bacteriophages, and phage therapy is started only after pathogen susceptibility has been confirmed in vitro. Notably, the pathogen is stored until the end of treatment to test wound fluid with the bacteriophage on day four or five after one-stage revision surgery, with mandatory bacteriological examination of the wound fluid.

The aim of this study was to compare the outcomes of two PJI treatment variants: combination phage/antibiotics therapy versus etiotropic systemic and local antibiotic therapy. Given our results, the efficacy of the antibiotic–bacteriophage combination therapy was one third higher in terms of the PJI relapse-free rate than a typical antibiotic regimen in these patients (difference = 31.9%; 95% CI for difference = 0.0443–0.5503), which resulted in an eight-fold decrease in the rate of PJI relapses in the SG compared to that in the CG (Fisher’s two-sided exact test *p*-value = 0.021), being eight (36.4%) cases in the CG versus one (4.5%) case in the SG, and a 12-fold lower risk of relapse in the SG compared to that in the CG.

We can compare our results on the 95.5% response rate to treatment (95% CI = 0.7511–0.9976) using phage-antibiotic combination therapy in the SG only with systematic reviews published in 2020–2021, by Clarke et al. [26] and Walter et al. [27], on the use of bacteriophages in the treatment of PJI. Study [26] reviewed seventeen reports, representing the treatment of 277 patients, with an efficacy estimate that revealed that 93.1% (*n* = 258/277) of patients achieved a clinical resolution, 3.3% (*n* = 9/277) showed an improvement, and 3.6% (*n* = 10/277) showed no improvement [26]. Review [27], involving 11 studies (eight case reports and three case studies, of which ten studies used bacteriophages in combination with antibiotics) with a total of 23 patients, showed that the complete eradication of infection over at least 7 months was achieved in 18 patients (78.3%); in two cases, there was a germ change [31], and a fistula persisted in one patient [50]; in two case studies, phage therapy did not preserve the extremities [51,52]. These results are unambiguously better than treatment of PJI with antibiotics only, the success rate of which in treatment of hip PJI with antibiotics/DAIR is 58–60.9% [48,49], in the latter case, given a daptomycin dose of 8 mg/kg [49]. In our study, the eradication rate, considered as a 12-month relapse-free period, in the CG (receiving antibiotics alone) was slightly higher and equals to 63.6% (95% CI = 0.4083–0.8198).

Importantly, the use of phage therapy does not guarantee complete infection eradication [27], which was also noted in our study: monitoring the efficacy of phage-antibiotic combination therapy at the surgical hospital for the first 12 ± 2 days after surgery revealed a germ change in one of 23 patients from the SG on day four of phage therapy (MRSE and *P. mirabilis* were detected). Microbiological investigation did not reveal any staphylococcal phage in wound fluid, which possibly indicated elimination of the initial *Staphylococcus* strain and occurrence of *P. mirabilis,* which was previously “hidden” in the infection site. This required switching from the combined phage/antibiotics therapy to treatment with antibiotics for up to 12 weeks. In other patients from the SG, neither the identified pathogen, which caused PJI, nor any additional infectious agent were found. 

An analysis of the phage titer in accordance with our proposed method [42] for monitoring the microbiological efficacy of bacteriophages in the treatment of PJI, showed that bacteriophage contents in wound fluid were 10^2^–10^4^ PFU/mL within 4 ± 1 days after the onset of phage therapy (the concentration of bacteriophages administered daily to patients through drains was therapeutic and amounted to at least 10^5^ PFU/mL). For this reason, phage therapy was considered effective and continued for 12 ± 2 days after surgery, given changes in the levels of the monitored inflammation markers ESR and CRP in samples from patients, until healing of a post-operative wound by primary intention.

In this study, we assessed the inhibition of infection based on changes in the acute phase reaction markers, ESR and CRP, as additional criteria for PJI [35]. Changes in the parameters monitored in the study indicated similar changes in the ESR and CRP values in the patient groups over time: a sharp increase by the third day of observation, followed by a progressive decrease in the CRP content in the SG, with the ESR level remaining unchanged after surgical treatment in both groups. An increase in the levels of ESR and CRP apparently reflects not only the patient’s response to surgery, but also the lytic activity of bacteriophages against the PJI pathogen. Usually, massive bacterial lysis after phage application is accompanied by the release of significant amounts of bacterial toxins, and provokes a strong acute phase response of the immune system. Whereas the ESR level can vary due to many different factors, including the patient’s gender and age, the CRP level is not affected by any of the known factors, except for the presence and severity of infectious inflammation, such as osteomyelitis and periprosthetic infection [53]. The CRP plasma’s half-life and catabolism level are controlled only by its synthesis, which, in turn, depends on the presence and severity of the infectious process [53].

Only two patients had an increase in temperature after the administration of phages, and the temperature returned to normal without additional treatment. This adverse drug reaction (ADR) can be explained by bacterial toxemia due to massive bacterial lysis during phage therapy. Otherwise, all other patients would also have had such an ADR after phage administration if the phage preparation had been insufficiently purified.

The hypothesis about the association between an increase in CRP level and the lytic activity of phages in the SG is also supported by a normalization of the CRP values on day 12 ± 2, and the absence of extremely high CRP values (outliers) in the SG on days 7 ± 1 and 12 ± 2, whereas such CRP values were recorded in several patients in the CG. The obtained data also indicated substantial differences in the CRP levels on day 3 ± 1 and a two-fold difference trend in CRP levels on day 7 ± 1 for the SG and CG, respectively. The faster, and continuous, decrease in the median and especially the third quartile values of CRP in the SG compared to the CG indicate a more effective elimination of PJI pathogens in the SG. Otherwise, the median CRP values in the patient groups on days 3 ± 1 and 7 ± 1 after surgery would be the same, and would reflect the usual acute phase response of the patient’s body to surgery. Such results can be explained by the possible phage-antibiotic synergy, which previously was described as a promising therapeutic effect in several in vitro experiments, in vivo animal model studies, and in a few clinical studies [31,54,55,56].

It is worth noting that in some cases, phage-antibiotic interactions can be antagonistic, depending on the class of antibiotic and the characteristics of the phage life cycle. So, the type of phage-antibiotic interaction should be checked before the use of phages with antibiotics, taking into account the antibiogram data and phage peculiarities. Additionally, the most effective combination of antibiotic and bacteriophage can be tested in such experiments.

Another important factor is that the antibiotics used at the inpatient stage of treatment in the SG are not biofilm-penetrating. Although we did not analyze microbial strains for their ability to form biofilm, the possibility of biofilm formation cannot be completely ruled out, especially given the duration of infection in the studied patients before revision surgery. In this case, microorganisms present in various biofilm layers can be affected only by bacteriophages possessing special enzymes to penetrate the biofilm, destroy its lipopolysaccharide structure [22,57,58,59,60,61,62], and eliminate persistent bacteria inside the biofilm [63].

Notably, our study had two limitations related to the study design: no randomization and no placebo control. Such drawbacks often become the subject of discussion in relation to surgical methods of treatment [64,65,66]. Indeed, it is far from always possible to use placebo control and randomization in surgical patients, both for ethical and clinical reasons. Similarly, this also applies to the present study, since the administration of such patients with PJI with a less effective treatment, or a “placebo”, may have led to a severe infectious process with devastating consequences, radically affecting the patient’s quality of life. In addition, studies conducted on relatively larger groups of patients would be of particular interest, since they would allow the evaluation of the PJI phage-antibiotic treatment efficacy to be more precise and allow the extrapolation of the obtained results to large populations of subjects.

## 5. Conclusions

The obtained results indicate that combination phage-antibiotic therapy is more effective than conventional antibiotic therapy and provides a significant advantage, with a 95.5% response rate to treatment. To achieve the best results in PJI patients, the phage titer in wound fluids should be constantly monitored. An important requirement for the use of phage preparations in the treatment of PJI is their mandatory combination with selected etiotropic antibiotics at the necessary doses and appropriate duration of their use. We suppose that simultaneous exposure of bacteria to two antibacterial agents with different natures (antibiotic and phage) complicates the emergence of persisters, resistant to both antimicrobials. Further prospective investigations are required to increase the sample size in PJI patients and evaluate the long-term outcomes of phage therapy. 

## Figures and Tables

**Table 1:** Patient distribution based on type and time of PJI manifestation according to classification of Tsukayama et al. [34] in both groups (ITT population).

Patient Groups	Early Post-Operative Infection	Late Chronic Infection	Acute Hematogenous Infection
Study group (*n* = 23)	5	13	5
Comparator group (*n* = 22)	6	12	4

**Table 2:** Etiology of PJI in both groups (ITT population).

Patient Groups	Number of Patients, *n* (%)					
	*S. epidermidis* (MSSE)	*S. epidermidis* (MRSE)	*S. aureus* (MSSA)	*S. haemolyticus* (MSSH)	*S. aureus* (MRSA)	Total per Group
Study group (SG)	8 (34.8%)	6 (26.1%)	8 (34.8%)	0 (0.0%)	1 (4.3%)	23 (100.0%)
Comparator group (CG)	4 (18.2%)	3 (13.6%)	13 (59.1%)	2 (9.1%)	0 (0.0%)	22 (100.0%)
Total	12 (26.7%)	9 (20.0%)	21 (46.7%)	2 (4.4%)	1 (2.2%)	45 (100.0%)

**Table 3:** Statistics for 12-month PJI relapse rate (primary endpoint, PP population).

		Patient Groups		Total
		Study Group	Comparator Group	
PJI relapse within 12 months	No	21 (95.5%)	14 (63.6%)	35 (79.5%)
	Yes	1 (4.5%)	8 (36.4%)	9 (20.5%)
Total		22 (100%)	22 (100%)	44 (100%)

**Table 4:** PJI relapse within 12 months (primary endpoint) odds ratio and risk estimation (population PP).

	Value	95% Confidence Interval	
		Lower Bound	Upper Bound
Odds Ratio for PJI relapse within 12 months (Yes/No)	0.083	0.009	0.742
Relative risk for PJI relapse within 12 months (SG)	0.185	0.029	1.199
Relative risk for PJI relapse within 12 months (CG)	2.222	1.393	3.545
Number of Valid Cases	44		

**Table 5:** Descriptive statistics for CRP (mg/L) and ESR (mm/hour) values during PJI treatment after revision surgery (ITT population).

		Mean	Standard Deviation	Maximum	Minimum	Median	Percentile 25	Percentile 75	Number of Patients
Baseline CRP	SG	43.5	43.95	167.45	3.19	29.8	13.62	56.40	23
	CG	37.7	44.35	196.08	1.60	24.6	14.84	36.78	22
CRP on day 3 ± 1	SG	104.9	59.65	236.70	22.38	86.8	57.99	158.66	23
	CG	57.5	30.32	124.21	22.76	44.1	34.40	69.32	22
CRP on day 7 ± 1	SG	45.3	24.95	87.45	8.44	45.5	20.57	56.75	22
	CG	42.8	49.61	162.44	4.02	23.5	10.82	53.44	22
CRP on day 12 ± 2	SG	21.4	12.86	46.75	1.95	17.2	13.61	28.85	22
	CG	39.9	60.02	250.56	1.97	16.0	9.06	46.22	22
Baseline ESR	SG	56.6	28.36	101	2	54.0	34.00	82.00	23
	CG	44.2	33.49	115	5	39.0	16.00	65.00	22
ESR on day 3 ± 1	SG	74.2	24.91	116	37	82.0	49.00	95.00	23
	CG	47.3	25.31	107	14	42.0	28.00	59.00	22
ESR on day 7 ± 1	SG	82.2	17.77	109	40	85.0	75.00	96.00	22
	CG	52.3	30.10	127	14	47.0	30.00	65.00	22
ESR on day 12 ± 2	SG	70.4	18.21	101	31	70.5	62.00	82.00	22
	CG	47.9	26.17	108	11	41.0	32.00	60.00	22

**Table 6:** Phage titer in the wound fluid obtained from patients of the study group (ITT population).

Patient Number	Pathogen in Wound Fluid on Day 4 ± 1 (Yes/No)	Identified Pathogen	Phage Titer
89457	No		1.8 × 10^2^ PFU/mL
89730	No		2.1 × 10^2^ PFU/mL
89866	No		2.3 × 10^3^ PFU/mL
90491	No		1.9 × 10^4^ PFU/mL
90783	No		1.8 × 10^3^ PFU/mL
87408	No		2.2 × 10^2^ PFU/mL
90660	No		2.3 × 10^2^ PFU/mL
94252	No		1.7 × 10^4^ PFU/mL
93001	No		2.8 × 10^3^ PFU/mL
95127	No		1.8 × 10^4^ PFU/mL
96583	No		2.1 × 10^2^ PFU/mL
97182	Yes	MRSE and *P. mirabilis*	0 (zero) PFU/mL
97604	No		1.6 × 10^3^ PFU/mL
92082	No		2.1 × 10^3^ PFU/mL
85770	No		1.9 × 10^4^ PFU/mL
29765	No		2.4 × 10^2^ PFU/mL
78175	No		2.2 × 10^2^ PFU/mL
86917	No		1.8 × 10^3^ PFU/mL
107497	No		1.7 × 10^3^ PFU/mL
86966	No		1.9 × 10^2^ PFU/mL
108819	No		2.1 × 10^4^ PFU/mL
116277	No		1.8 × 10^3^ PFU/mL
116463	No		2.3 × 10^2^ PFU/mL

## Data Availability

The data presented in this study are available on request from the corresponding author. The data are not publicly available due to ethical reasons.

## References

[1] Rosteius T., Jansen O., Fehmer T., Baecker H., Citak M., Schildhauer T.A., Geßmann J. (2018). Evaluating the Microbial Pattern of Periprosthetic Joint Infections of the Hip and Knee. J. Med. Microbiol..

[2] Bozhkova S.A., Kasimova A.R., Tikhilov R.M., Polyakova E.M., Rukina A.N., Shabanova V.V., Liventsov V.N. (2018). Adverse Trends in the Etiology of Orthopedic Infection: Results of 6-Year Monitoring of the Structure and Resistance of Leading Pathogens. Traumatol. Orthop. Russ..

[3] Mullen A., Wieland H.J., Wieser E.S., Spannhake E.W., Marinos R.S. (2017). Perioperative Participation of Orthopedic Patients and Surgical Staff in a Nasal Decolonization Intervention to Reduce Staphylococcus Spp Surgical Site Infections. Am. J. Infect. Control.

[4] Fink B., Schuster P., Braun R., Tagtalianidou E., Schlumberger M. (2020). The Diagnostic Value of Routine Preliminary Biopsy in Diagnosing Late Prosthetic Joint Infection after Hip and Knee Arthroplasty. Bone Jt. J..

[5] Pavlov V.V., Petrova N.V., Sheraliev T.U. (2019). Two-Stage Treatment of Periprostetic Infection: Mid-Term Results. Traumatol. Orthop. Russ..

[6] Antonelli B., Chen A.F. (2019). Reducing the Risk of Infection after Total Joint Arthroplasty: Preoperative Optimization. Arthroplasty.

[7] Pichkhadze I.M., Kuz’menkov K.A., Zhadin A.V., Tsiskarashvili A.V., Pichkhadze E.I., Daneliya L.M., Rekvava G.R., Shulashov B.N., Pichkhadze I.M., Kuzmenkov K.A. (2009). Treatment of Patients with Pyo-Inflammatory Complications after Hip Replacement. N.N. Priorov J. Traumatol. Orthop..

[8] Zimmerli W., Trampuz A., Ochsner P.E. (2004). Prosthetic-Joint Infections. N. Engl. J. Med..

[9] De la Fuente-Núñez C., Reffuveille F., Fernández L., Hancock R.E. (2013). Bacterial Biofilm Development as a Multicellular Adaptation: Antibiotic Resistance and New Therapeutic Strategies. Curr. Opin. Microbiol..

[10] Afinogenova A.G. (2011). Microbial Biofilms of Wounds: Status of the Issue. Traumatol. Orthop. Russ..

[11] Jaffe D., Costales T., Greenwell P., Christian M., Henn R. (2017). Methicillin-Resistant Staphylococcus Aureus Infection Is a Risk Factor for Unplanned Return to the Operating Room in the Surgical Treatment of a Septic Knee. J. Knee Surg..

[12] Janz V., Trampuz A., Perka C.F., Wassilew G.I. (2017). Reduced Culture Time and Improved Isolation Rate through Culture of Sonicate Fluid in Blood Culture Bottles. THC.

[13] Kurd M.F., Ghanem E., Steinbrecher J., Parvizi J. (2010). Two-Stage Exchange Knee Arthroplasty: Does Resistance of the Infecting Organism Influence the Outcome?. Clin. Orthop. Relat. Res..

[14] Leung F., Richards C.J., Garbuz D.S., Masri B.A., Duncan C.P. (2011). Two-Stage Total Hip Arthroplasty: How Often Does It Control Methicillin-Resistant Infection?. Clin. Orthop. Relat. Res..

[15] Kuo F.-C., Yen S.-H., Peng K.-T., Wang J.-W., Lee M.S. (2015). Methicillin-Resistant Staphylococcal Periprosthetic Joint Infections Can Be Effectively Controlled by Systemic and Local Daptomycin. BMC Infect. Dis..

[16] Chang Y.-J., Lee M.S., Lee C.-H., Lin P.-C., Kuo F.-C. (2017). Daptomycin Treatment in Patients with Resistant Staphylococcal Periprosthetic Joint Infection. BMC Infect Dis..

[17] Vlassov V.V., Tikunova N.V., Morozova V.V. (2020). Bacteriophages as Therapeutic Preparations: What Restricts Their Application in Medicine. Biochemistry.

[18] Sillankorva S., Neubauer P., Azeredo J. (2010). Phage Control of Dual Species Biofilms of Pseudomonas Fluorescens and Staphylococcus Lentus. Biofouling.

[19] Sillankorva S., Oliveira R., Vieira M.J., Azeredo J. (2008). Real-Time Quantification of Pseudomonas Fluorescens Cell Removal from Glass Surfaces Due to Bacteriophage ΦS1 Application. J. Appl. Microbiol..

[20] Hanlon G.W., Denyer S.P., Olliff C.J., Ibrahim L.J. (2001). Reduction in Exopolysaccharide Viscosity as an Aid to Bacteriophage Penetration through *Pseudomonas Aeruginosa* Biofilms. Appl. Environ. Microbiol..

[21] Hughes K.A., Sutherland I.W., Clark J., Jones M.V. (1998). Bacteriophage and Associated Polysaccharide Depolymerases—Novel Tools for Study of Bacterial Biofilms. J. Appl. Microbiol..

[22] Hughes K.A., Sutherland I.W., Jones M.V. (1998). Biofilm Susceptibility to Bacteriophage Attack: The Role of Phage-Borne Polysaccharide Depolymerase. Microbiology.

[23] Sutherland I.W., Hughes K.A., Skillman L.C., Tait K. (2004). The Interaction of Phage and Biofilms. FEMS Microbiol. Lett..

[24] Lu T.K., Collins J.J. (2007). Dispersing Biofilms with Engineered Enzymatic Bacteriophage. Proc. Natl. Acad. Sci. USA.

[25] Tkhilaishvili T., Wang L., Tavanti A., Trampuz A., Di Luca M. (2020). Antibacterial Efficacy of Two Commercially Available Bacteriophage Formulations, Staphylococcal Bacteriophage and PYO Bacteriophage, Against Methicillin-Resistant Staphylococcus Aureus: Prevention and Eradication of Biofilm Formation and Control of a Systemic Infection of Galleria Mellonella Larvae. Front. Microbiol..

[26] Clarke A., De Soir S., Jones J. (2020). The Safety and Efficacy of Phage Therapy for Bone and Joint Infections: A Systematic Review. Antibiotics.

[27] Walter N., Deng L., Brochhausen C., Alt V., Rupp M. (2022). Behandlung von Knochen- und Protheseninfektionen mit Bakteriophagen: Ein systematisches Review. Orthopäde.

[28] Międzybrodzki R., Borysowski J., Weber-Dąbrowska B., Fortuna W., Letkiewicz S., Szufnarowski K., Pawełczyk Z., Rogóż P., Kłak M., Wojtasik E. (2012). Clinical Aspects of Phage Therapy. Advances in Virus Research.

[29] Ferry T., Boucher F., Fevre C., Perpoint T., Chateau J., Petitjean C., Josse J., Chidiac C., L’hostis G., Leboucher G. (2018). Innovations for the Treatment of a Complex Bone and Joint Infection Due to XDR Pseudomonas Aeruginosa Including Local Application of a Selected Cocktail of Bacteriophages. J. Antimicrob. Chemother..

[30] Rogóż P., Amanatullah D.F., Międzybrodzki R., Manasherob R., Tikunova N.V., Weber-Dąbrowska B., Fortuna W., Letkiewicz S., Górski A., Górski A., Międzybrodzki R., Borysowski J. (2019). Phage Therapy in Orthopaedic Implant-Associated Infections. Phage Therapy: A Practical Approach.

[31] Patey O., McCallin S., Mazure H., Liddle M., Smithyman A., Dublanchet A. (2018). Clinical Indications and Compassionate Use of Phage Therapy: Personal Experience and Literature Review with a Focus on Osteoarticular Infections. Viruses.

[32] Doub J.B., Ng V.Y., Johnson A.J., Slomka M., Fackler J., Horne B., Brownstein M.J., Henry M., Malagon F., Biswas B. (2020). Salvage Bacteriophage Therapy for a Chronic MRSA Prosthetic Joint Infection. Antibiotics.

[33] Samokhin A.G., Fedorov E.A., Kozlova Y.N., Tikunova N.V., Pavlov V.V., Morozova V.V., Kretien S.O. (2016). Application of the Lytic Bacteriophages during Surgical Treatment of the Periprosthetic Infection of the Hip Joint Endoprosthesis (Pilot Study). MPSE.

[34] Tsukayama D.T., Estrada R., Gustilo R.B. (1996). Infection after Total Hip Arthroplasty. A Study of the Treatment of One Hundred and Six Infections*. J. Bone Jt. Surg..

[35] Parvizi J., Zmistowski B., Berbari E.F., Bauer T.W., Springer B.D., Della Valle C.J., Garvin K.L., Mont M.A., Wongworawat M.D., Zalavras C.G. (2011). New Definition for Periprosthetic Joint Infection: From the Workgroup of the Musculoskeletal Infection Society. Clin. Orthop. Relat. Res..

[36] Paprosky W., Lawrence J., Cameron H. (1990). Femoral Defect Classification: Clinical Application. Orthop. Rev..

[37] Paprosky W.G., Perona P.G., Lawrence J.M. (1994). Acetabular Defect Classification and Surgical Reconstruction in Revision Arthroplasty. J. Arthroplast..

[38] Parvizi J., Gehrke T., Chen A.F. (2013). Proceedings of the International Consensus on Periprosthetic Joint Infection. Bone Jt. J..

[39] Parvizi J., Fassihi S.C., Enayatollahi M.A. (2016). Diagnosis of Periprosthetic Joint Infection Following Hip and Knee Arthroplasty. Orthop. Clin. N. Am..

[40] Kutter E., Clokie M.R.J., Kropinski A.M. (2009). Phage Host Range and Efficiency of Plating. Bacteriophages.

[41] Samokhin A.G., Kozlova J.N., Korneev D.V., Taranov O.S., Fedorov E.A., Pavlov V.V., Morozova V.V., Tikunova N.V. (2018). Experimental Study of the Antibacterial Activity of the Lytic Staphylococcus Aureus Bacteriophage Ph20 and Lytic Pseudomonas Aeruginosa Bacteriophage Ph57 during Modelling of Its Impregnation into Poly(Methylmetacrylate) Orthopedic Implants (Bone Cement). Ann. RAMS.

[42] Pavlov V.V., Samokhin A.G., Fedorov E.A., Prokhorenko V.M., Kretien S.O., Kozlova Y.N., Tikunova N.V., Morozova V.V. Method of Evaluating Efficiency of Phagotherapy in Treatment of Infections Diseases. Patent RU2624511C1. https://patents.google.com/patent/RU2624511C1/en.

[43] Appelmans R. (1921). Le Dosage Du Bacteriophage. Compt. Rend. Soc. Biol..

[44] Renz N., Trampuz A. (2015). Pocket Guide to Diagnosis & Treatment of Periprosthetic Joint Infection.

[45] Newcombe R.G. (1998). Two-Sided Confidence Intervals for the Single Proportion: Comparison of Seven Methods. Statist. Med..

[46] Wilson E.B. (1927). Probable Inference, the Law of Succession, and Statistical Inference. J. Am. Stat. Assoc..

[47] Chow S.-C., Shao J., Wang H., Lokhnygina Y. (2017). Sample Size Calculations in Clinical Research. Chapman & Hall/CRC Biostatistics Series.

[48] Lora-Tamayo J., Euba G., Cobo J., Horcajada J.P., Soriano A., Sandoval E., Pigrau C., Benito N., Falgueras L., Palomino J. (2016). Short- versus Long-Duration Levofloxacin plus Rifampicin for Acute Staphylococcal Prosthetic Joint Infection Managed with Implant Retention: A Randomised Clinical Trial. Int. J. Antimicrob. Agents.

[49] Byren I., Rege S., Campanaro E., Yankelev S., Anastasiou D., Kuropatkin G., Evans R. (2012). Randomized Controlled Trial of the Safety and Efficacy of Daptomycin versus Standard-of-Care Therapy for Management of Patients with Osteomyelitis Associated with Prosthetic Devices Undergoing Two-Stage Revision Arthroplasty. Antimicrob. Agents Chemother..

[50] Ferry T., Kolenda C., Batailler C., Gustave C.-A., Lustig S., Malatray M., Fevre C., Josse J., Petitjean C., Chidiac C. (2020). Phage Therapy as Adjuvant to Conservative Surgery and Antibiotics to Salvage Patients With Relapsing S. Aureus Prosthetic Knee Infection. Front. Med..

[51] Ferry T., Batailler C., Petitjean C., Chateau J., Fevre C., Forestier E., Brosset S., Leboucher G., Kolenda C., Laurent F. (2020). The Potential Innovative Use of Bacteriophages Within the DAC^®^ Hydrogel to Treat Patients With Knee Megaprosthesis Infection Requiring “Debridement Antibiotics and Implant Retention” and Soft Tissue Coverage as Salvage Therapy. Front. Med..

[52] Vogt D., Sperling S., Tkhilaishvili T., Trampuz A., Pirnay J.-P., Willy C. (2017). „Beyond antibiotic therapy“—Zukünftige antiinfektiöse Strategien—Update 2017. Unfallchirurg.

[53] Jurado R.L. (2001). Why Shouldn’t We Determine the Erythrocyte Sedimentation Rate?. Clin. Infect. Dis..

[54] Torres-Barceló C., Arias-Sánchez F.I., Vasse M., Ramsayer J., Kaltz O., Hochberg M.E. (2014). A Window of Opportunity to Control the Bacterial Pathogen Pseudomonas Aeruginosa Combining Antibiotics and Phages. PLoS ONE.

[55] Torres-Barceló C., Hochberg M.E. (2016). Evolutionary Rationale for Phages as Complements of Antibiotics. Trends Microbiol..

[56] Diallo K., Dublanchet A. (2022). Benefits of Combined Phage–Antibiotic Therapy for the Control of Antibiotic-Resistant Bacteria: A Literature Review. Antibiotics.

[57] Tait K., Skillman L.C., Sutherland I.W. (2002). The Efficacy of Bacteriophage as a Method of Biofilm Eradication. Biofouling.

[58] Chaudhry W.N., Concepción-Acevedo J., Park T., Andleeb S., Bull J.J., Levin B.R. (2017). Synergy and Order Effects of Antibiotics and Phages in Killing Pseudomonas Aeruginosa Biofilms. PLoS ONE.

[59] Kumaran D., Taha M., Yi Q., Ramirez-Arcos S., Diallo J.-S., Carli A., Abdelbary H. (2018). Does Treatment Order Matter? Investigating the Ability of Bacteriophage to Augment Antibiotic Activity against Staphylococcus Aureus Biofilms. Front. Microbiol..

[60] Tkhilaishvili T., Winkler T., Müller M., Perka C., Trampuz A. (2019). Bacteriophages as Adjuvant to Antibiotics for the Treatment of Periprosthetic Joint Infection Caused by Multidrug-Resistant Pseudomonas Aeruginosa. Antimicrob. Agents Chemother..

[61] Necel A., Bloch S., Topka-Bielecka G., Janiszewska A., Łukasiak A., Nejman-Faleńczyk B., Węgrzyn G. (2022). Synergistic Effects of Bacteriophage VB_Eco4-M7 and Selected Antibiotics on the Biofilm Formed by Shiga Toxin-Producing Escherichia Coli. Antibiotics.

[62] Łusiak-Szelachowska M., Weber-Dąbrowska B., Górski A. (2020). Bacteriophages and Lysins in Biofilm Control. Virol. Sin..

[63] Tkhilaishvili T., Lombardi L., Klatt A.-B., Trampuz A., Di Luca M. (2018). Bacteriophage Sb-1 Enhances Antibiotic Activity against Biofilm, Degrades Exopolysaccharide Matrix and Targets Persisters of Staphylococcus Aureus. Int. J. Antimicrob. Agents.

[64] Das A.K. (2011). Randomised Clinical Trials in Surgery: A Look at the Ethical and Practical Issues. Indian J. Surg..

[65] Savulescu J., Wartolowska K., Carr A. (2016). Randomised Placebo-Controlled Trials of Surgery: Ethical Analysis and Guidelines. J. Med. Ethics.

[66] Cooper J.D. (2010). Randomized Clinical Trials for New Surgical Operations: Square Peg in a Round Hole?. J. Thorac. Cardiovasc. Surg..

